# Ecological impacts of time-variable exposure regimes to the fungicide azoxystrobin on freshwater communities in outdoor microcosms

**DOI:** 10.1007/s10646-012-0856-9

**Published:** 2012-01-26

**Authors:** Mazhar Iqbal Zafar, J. Dick M. Belgers, Rene P. A. Van Wijngaarden, Arriënne Matser, Paul J. Van den Brink

**Affiliations:** 1 Department of Aquatic Ecology and Water Quality Management, Wageningen University, P.O. Box 47, 6700 AA Wageningen, The Netherlands; 2 Alterra, Wageningen University and Research Centre, P.O. Box 47, 6700 AA Wageningen, The Netherlands

**Keywords:** Pesticides, Microcosm, Invertebrates, Time-variable exposure, Risk assessment, Azoxystrobin

## Abstract

This paper evaluates the effects of different time-varying exposure patterns of the strobilurin fungicide azoxystrobin on freshwater microsocosm communities. These exposure patterns included two treatments with a similar peak but different time-weighted average (TWA) concentrations, and two treatments with similar TWA but different peak concentrations. The experiment was carried out in outdoor microcosms under four different exposure regimes; (1) a continuous application treatment of 10 μg/L (CAT_10_) for 42 days (2), a continuous application treatment of 33 μg/L (CAT_33_) for 42 days (3), a single application treatment of 33 μg/L (SAT_33_) and (4) a four application treatment of 16 μg/L (FAT_16_), with a time interval of 10 days. Mean measured 42-d TWA concentrations in the different treatments were 9.4 μg/L (CAT_10_), 32.8 μg/L (CAT_33_), 14.9 μg/L (SAT_33_) and 14.7 μg/L (FAT_16_). Multivariate analyses demonstrated significant changes in zooplankton community structure in all but the CAT_10_ treated microcosms relative to that of controls. The largest adverse effects were reported for zooplankton taxa belonging to Copepoda and Cladocera. By the end of the experimental period (day 42 after treatment), community effects were of similar magnitude for the pulsed treatment regimes, although the magnitude of the initial effect was larger in the SAT_33_ treatment. This indicates that for long-term effects the TWA is more important for most zooplankton species in the test system than the peak concentration. Azoxystrobin only slightly affected some species of the macroinvertebrate, phytoplankton and macrophyte assemblages. The overall no observed ecologically adverse effect concentrations (NOEAEC) in this study was 10 µg/L.

## Introduction

In the European Union (EU), ecological risk assessment of pesticides follows a tiered approach, which is laid down in the European pesticide regulation (European Commission 2009) and underlying guidance documents (European Commission 2002). For non-target aquatic organisms, higher-tier risk assessments traditionally have incorporated the results of additional laboratory and semi-field experiments evaluating a range of pesticide concentrations that have a single or repeated pulse exposure, or that are held constant for a short period of time. It has been recognised, however, that under field conditions, aquatic non-target organisms may be exposed to fluctuating concentrations of pesticide contaminants (Reinert et al. 2002) and consequently, in recent years, more attention has been paid to pulsed or intermittent exposure scenarios. Prediction of effects of pulsed or intermittent exposure on populations is becoming an important issue in ecotoxicology (Boesten et al. 2007; Van den Brink 2008). This issue was highlighted in a recent EU ELINK workshop (Brock et al. 2010), that resulted in recommendations for addressing time-variable exposures in aquatic risk assessment for pesticides, which developed guidance on when to use the peak or the time-weighted average (TWA) concentrations.

The present study evaluated the effects of azoxystrobin, a broad-spectrum, systemic fungicide belonging to the group β-methoxyacrylate strobilurins, with a biochemical mode of action that acts on respiration by inhibiting electron transport from cytochrome *B* to cytochrome *C*. It was first marketed in 1996 and has since then been registered worldwide for use on a wide range of crops (Bartlett et al. 2002). A range of laboratory, field, or semi-field toxicity data have been published for the fungicide azoxystrobin (Maltby et al. 2009; Warming et al. 2009; Cole et al. 2000, see EFSA 2009 for a summary of this confidential report; Gustafsson et al. 2010).

Fungicides can be toxic to a wide array of aquatic non-target organisms, and may affect the structure and function of biological communities (Maltby et al. 2009; Van den Brink et al. 2000; Slijkerman et al. 2004). According to our knowledge, no other information than that published in Cole et al. (2000) and Gustafsson et al. (2010) is available on the effects of strobilurin fungicides on aquatic systems of higher biological complexity than single species tests. Gustafsson et al. (2010) investigated the ecological effects of the fungicide azoxystrobin in outdoor brackish water microcosms and found that azoxystrobin is toxic to brackish water copepods at considerably lower concentration (≤3 μg/L) than previously reported for single species tests performed with freshwater crustaceans. Cole et al. (2000) tested the effects of a commercial formulation (YF9246, a 250 g/L suspension concentrate) of azoxystrobin on freshwater microcosms and found that zooplankton were more sensitive than other endpoints, with transient effects reported at 10 μg/L.

The present study was initiated to investigate, using azoxystrobin, which concentration profile, be it TWA or peak, is more appropriate for assessing the longer-term aquatic risks of this pesticide. This followed a similar microcosm study with the insecticide chlorpyrifos, which concluded that for most species, but not for all, the TWA concentration was more important than the peak concentration in explaining the longer-term effects (Zafar et al. 2011). The aim of the present study was to compare the effects of four different exposure regimes (two chronic, maintained exposure profiles, one repeated pulsed, and one single pulsed exposure regime) for the fungicide azoxystrobin. The high chronic and single pulse regimes had similar peak but different TWA concentrations, while the two pulsed regimes had different peak but similar TWA concentrations. The TWA concentrations of the two pulsed exposure regimes were intermediate relative to the two chronic regimes.

## Materials and methods

### Experimental design

Sixteen outdoor microcosms (diameter 1.8 m, total depth 0.8 m, water depth 0.5 m, water volume ca. 1,270 L) were used in the experiment. The microcosms were located at the Sinderhoeve Experimental Station (www.sinderhoeve.org) in Renkum near Wageningen, The Netherlands, and were lined with a watertight non-toxic layer of black polyethylene. Each microcosm was initially established with an 8 cm layer of sediment (fine clay) from a mesotrophic lake (dominated by the aquatic plants *Elodea nuttallii* and *Chara* sp.) and then filled with water, taken from the experimental station’s water supply basin.

In the preparatory phase, one hundred shoots of *Elodea nuttallii* were planted on 75% of the sediment surface of each microcosm. In addition, other macrophytes (*Eleocharis acicularis*, *Spirodela polyrhiza*, *Potamogeton berchtoldii*, *Potamogeton pectinatus, Elodea canadensis, Potamogeton crispus* and *Ranunculus circinatus*) developed from diaspores in the sediment during the course of study. During the pre-treatment period (3 months approximately), phytoplankton, zooplankton and macroinvertebrates were collected from uncontaminated mesotrophic ditches situated at the Sinderhoeve Experimental Station, and Veenkampen, an experimental field site of Wageningen University, Wageningen, The Netherlands and introduced into the systems in order to develop a freshwater community characteristic for lentic, edge-of-field surface water. The macroinvertebrates introduced comprised several taxonomic groups and they were representatives of various trophic levels. Dominant species included crustaceans (*Asellus aquaticus*, *Gammarus pulex* and *Daphnia* sp.), insects (*Cloeon dipterum*, *Chaoborus* sp., *Plea minutissima*, Chironomidae, odonates and trichopterans), and the non-arthropods Hirudinea (*Erpobdella* sp.) and Gastropoda (*Valvata* sp.).

During the pre-treatment period all microcosms were interconnected by tubes and the water was circulated using a pump to achieve the development of a similar biocoenoses in the test systems. The circulation of water was stopped 3 weeks before the start of the experiment.

The microcosms were investigated over a period of 7 weeks. One week prior to the first applications, all biological endpoints were sampled once to establish pre-treatment conditions, followed by a post treatment period of approximately 6 weeks.

### Pesticide application and sampling

**Fig. 1:**
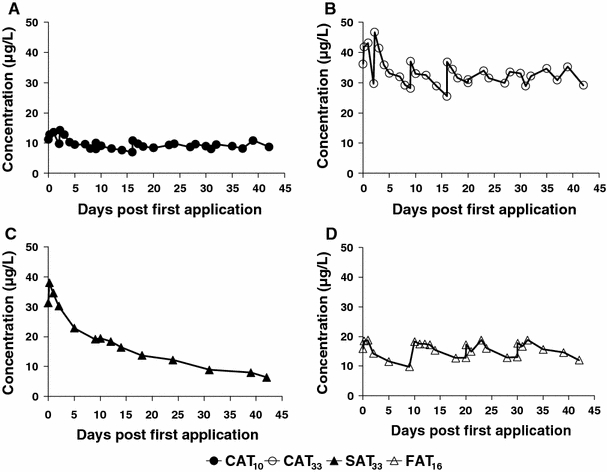
Dynamics of azoxystrobin concentrations in microcosms water during the 42 days period **a** continuous exposure of 10 μg/L, **b** continuous exposure of 33 μg/L, **c** single application and **d** four application treatments. The four applications took place on day 0, 10, 20 and 30

Azoxystrobin was provided by Syngenta Crop Protection AG, Switzerland as the formulated product AMISTAR^®^ (Fluid) a 250 g a.i./L soluble concentrate formulation). There were four intended treatment regimes: (1) a continuous application treatment (CAT) of 10 μg/L (CAT_10_) consisting of a continuous exposure to 10 μg a.i./L for 42 days (2) a continuous application treatment of 33 μg/L (CAT_33_) consisting of a continuous exposure to 33 μg a.i./L for 42 days (3) a single application treatment (SAT_33_) consisting of a single application of 33 μg a.i./L and (4) a four application treatment (FAT_16_) consisting of four applications, each achieving a peak of 16 μg a.i./L with a time interval of 10 days. The treatment levels of the SAT_33_ and FAT_16_ applications were based on the 42d-TWA of 15 μg a.i./L, which fell in between the chronic exposure regimes of CAT_10_ and CAT_33_. The concentrations in the chronic tests were kept constant between 80 and 120% of desired nominal concentrations by adding more azoxystrobin during exposure. To measure the exposure concentrations, water samples from all microcosm were collected regularly (see Fig. 1). In the continuous exposure treatments, sampling and analysis of azoxystrobin were performed every 1–2 days, with dosing as necessary to maintain the concentration. Approximately 1 h after the additional application, a water sample was taken and the concentration analysed as described below. Before application, concentrations in stock and dosing solutions were checked for establishing nominal initial concentrations. The first treatment day is referred to as day 0, the first sampling as day −7 while the post first treatment days run up to day 43.

The microcosms were randomly allocated to the different treatments. All treatments were performed in triplicate with four control replicates. Azoxystrobin was applied by pouring a defined volume of dosing solution into the microcosms. The control microcosms were treated with water only. The systems were gently stirred immediately after application to promote the mixing through the water column whilst avoiding any resuspension of sediment particles and disturbance of submerged macrophytes.

### Calculation of treatment level for time-variable exposures

Azoxystrobin was selected as a compound for this study, as it has a measured waterphase DT_50_ of 13 days in an outdoor aquatic microcosm (Jones and Lake 2000, see EFSA 2009 for a summary of this confidential report). In addition, with a log K_ow_ of 2.5 azoxystrobin would be expected to remain mainly in the water phase (Tomlin 2011). Due to its relatively slow dissipation, exposures would be expected to be moderate to long-term. The concentrations of azoxystrobin chosen were based on the 10 μg/L NOEAEC (no observed ecologically adverse effect concentration (effect class 2/slight effects)) derived from a single application to an outdoor pond microcosm study (Cole et al. 2000). More pronounced effects may be expected when this concentration is maintained. Therefore, the intention was for the concentration in CAT_10_ to be equal to the 42d-TWA in SAT_33_ and FAT_16_ (calculations according to Zafar et al. (2011)). However, azoxystrobin proved to be more persistent in our microcosms and consequently the TWA concentration of the SAT_33_ and FAT_16_ were 15 μg/L instead of 10 μg/L, and therefore this TWA concentration fell in between CAT_10_ and CAT_33_.

### Azoxystrobin analysis

The concentrations of azoxystrobin were determined in the water samples by taking depth-integrated water samples from the microcosms by means of stainless steel suction tubes connected to glass flasks (Schott bottle, 250 mL) using a vacuum pump. Approximately 100 mL of water were sampled from each microcosm in duplicate. Duplicate 2 mL samples from the 100 mL-water sample were transferred into 4-mL WISP vials (borosilicate) containing 2 mL of acetonitrile. The exact mass of water added was calculated by weighing the vials. The vials were closed with a cap and thoroughly shaken manually. A 2 mL high performance liquid chromatograpy (HPLC) vial was then filled with a portion of the sample, and was then sealed and analysed by Liquid chromatography-mass spectrometry with Triple Quadrupole systems (P2600 Agilent 6410 LC–MS/MS QQQ). The volume injected was 50 μL with an autosampler and the mobile phase (HPLC—water/acetonitrile; (50/50, V/V) was set at a flow rate of 1.0 mL/min. The analytical column used was an Agilent Zorbax Eclipse XDB-C18 (Diameter 4.6 mm; Length 150 mm; 5 μm). Column was set at temperature 40°C. Under these conditions, the retention time of azoxystrobin was approximately 2.40 min.

TWA concentrations of azoxystrobin were based on area under the curve (AUC) calculations, and the DT_50_ in SAT_33_ was estimated assuming first-order dissipation kinetics. Dissipation times were based on measurements for water samples above the limit of quantification (LOQ). The Level of Detection (LOD) and LOQ of the analysis were determined by adding a standard 0.01 μg/L of azoxystrobin in acetonitrile/water (v/v: 50/50) to each injection series. The concentration of this standard of 0.01 μg/L azoxystrobin was calculated from the calibration curve, while the standard itself was not part of this calibration curve. In total, this standard was injected 105 times, yielding an average concentration of 0.0208 μg/L, with a standard deviation (SD) of 0.0048 μg/L. The LOD in water sample was defined as 3 × SD (3 × 0.0048 = 0.015 μg/L), the LOQ as 10 × SD (10 × 0.0048 = 0.05 μg/L). The DT_50_ was calculated by means of linear regression using ln-transformed measured pesticide concentrations versus time.

### Macroinvertebrates

Artificial substrates, consisting of litter bags (see “Decomposition” section) and pebble baskets, were used to monitor the effects of azoxystrobin on the benthic macroinvertebrate assemblage. Two pebble baskets and two litter bags were placed on concrete tiles on the sediment in each microcosm 2 weeks before the initiation of the treatments in order to allow colonisation by macroinvertebrates (for a detailed description of methods see Brock et al. (1992)).

Macroinvertebrates were sampled five times from each microcosm at days −7, 3, 10, 17 and 43. Pebble baskets were gently retrieved using a net. The litter bags were collected by hand. The substrates were first washed in a container to remove invertebrates. The macroinvertebrates were identified and counted alive, and then released back into the their original microcosms. The animals were identified to the lowest practical taxonomic level. From each microcosm abundance of macroinvertebrates from pebble baskets and litter bags were pooled prior to analysis of the data.

### Phyto- and zooplankton sampling and identification

Zooplankton and phytoplankton were simultaneously sampled on days −5, 2, 9, 16, 23, 32 and 44 days by using a Perspex (Poly(methyl methacrylate)) tube (volume = 1.8 L). Depth-integrated water samples were collected from several spots in each microcosm until a bulk water sample of 12 L had been obtained in a bucket. From this bulk sample, 5 L was passed through a 55 μm mesh net to collect zooplankton. Another 5 L was passed through a 20 μm mesh net to collect phytoplankton, possibly missing the smaller phytoplankton taxa. The concentrated plankton samples were preserved with acetate buffered Lugol’s solution in a 100 mL sampling vial. The filtered water was returned into its original microcosm.

Cladocerans, copepods and ostracods (macro-zooplankton) were counted using a stereo microscope (Nikon SMZ-10, magnification 25×). Rotifers and copepod nauplii (micro-zooplankton) were quantified and identified with an inverted microscope (Carl Zeiss, Axiovert 10, magnification 100×), using a sub-sample of known volume. Rotifers and cladocerans were identified to the lowest practical taxonomic level (i.e., genus or species level), whereas copepods were identified to the suborder by classifying as calanoids or cyclopoids. A distinction was also made between nauplii and the more mature stages of the copepods.

Phytoplankton species composition was studied by counting the number of cells of a known volume which were identified to the lowest practical taxonomic level. Taxa and number of cells were based on a maximum of 200 observations, consisting of a series of 20–40 counting fields of a single cuvette under an inverted microscope (magnification 400×). Zooplankton and phytoplankton data were expressed as number of individuals per litre.

### Chlorophyll-*a*

Phytoplankton chlorophyll-*a* was sampled in parallel with the phyto- and zooplankton sampling. One litre of the remaining from the bulk 12-l sample was used to determine the amount of chlorophyll-*a* of the phytoplankton. Samples were concentrated through a 1.2 μm pore size Whatmann glass-fibre filter (GF/C; diameter 4.7 cm; Maidstone, UK) using a vacuum pump. The filters containing phytoplankton were transferred into Petri dishes, wrapped in aluminium foil, and stored in a freezer at a temperature of –70°C until analysis. After ethanol extraction of the pigments, measurements of chlorophyll-*a* content were performed using a HPLC with fluorescence detection (Webb et al. 1992).

As an estimate of periphytic algal biomass, chlorophyll-*a* was sampled on day −5 and on days 2, 9, 16, 23, 32 and 42. Periphyton was sampled from glass microscope slides (7.6 × 2.6 cm) that served as artificial substrates. The slides were positioned vertically in a stainless steel frame placed in the centre of all microcosm in the north–south position tied on a long rod, approximately 10 cm below the water surface of each microcosm, and incubated for 2 weeks. The placement of frame was kept the same in all test systems during whole experimental period. On each sampling day, 8 glass slides per microcosm (colonised for 14 days) were scraped visually clean with blades (Applo Ever-Sharp-Blades; Solingen-Germany) collecting the removed periphyton in tap water. New clean slides were then reintroduced in the microcosm. The chlorophyll-*a* content of the water periphyton solution was analysed as described above for the phytoplankton.

### Water quality parameters

Dissolved oxygen (DO), pH, electrical conductivity (EC) and temperature (T) were measured in each microcosms on days −5, 2, 9, 16, 23, 32 and 42 to detect possible changes in community metabolism. On sampling days, measurements were carried out in the morning just around the start of photoperiod, at approximately 25 cm below the water surface. h DO, pH and T were measured using a HQ40D multimeter (Hach-Lange, The Netherlands) and EC was measured with an Eijkelkamp 18.28 conductivity meter.

Alkalinity levels were determined in all microcosms prior to the initiation of the treatments (day −5) and at the end (day 43) of the experiment, using 100-mL water samples taken at a depth of 10 cm by titrating with 0.02 N HCl until a pH of 4.2 was reached (pH meter: WTW 323).

Additionally, the concentration of ammonia, nitrate, nitrite, total nitrogen, orthophosphate and total phosphate were measured in the control microcosms at the start of the experiment and in all microcosms at end of experiment (day 42). For this purpose, water samples (approximately 100 mL) were obtained from the filtered water (Whattman GF/C; 1.2 μm pore-size) collected for phytoplankton chlorophyll-*a* samples. These samples were transferred into 100-mL polyethylene flasks which were stored at below −18°C until analysis. Total soluble nitrogen, N–(NO_2_^−^ + NO_3_^−^), NH_4_^+^, ortho-phosphate and total phosphate were analysed using a Skalar 5100 Autoanalyser.

### Decomposition

Decomposition of particulate organic matter (POM) was determined using litter bags (Brock et al. 1982), containing *Populus* × *canadensis* (hybrid black poplar) leaves. In the decomposition assessment, a portion of 2 g dry weight (dried at 60°C) of leaves were enclosed in each litter bag. The litter bags were made from a glass Petri-dish (diameter: 11.6 cm), closed with a cover of stainless-steel wire (mesh size: 0.7 × 0.7 mm), in which two holes (diameter: 0.5 cm) were punched to give invertebrates access to the leaves.

In each microcosm, two litter bags were placed at the sediment surface in an almost upright position for a 2-week incubation period. At the end of the incubation period, litter bags were emptied into a white tray to separate POM from adhering sediment particles and macroinvertebrates by rinsing with tap water. After sampling, a new set of litterbags was incubated. Remaining organic plant material was dried in pre-weighted aluminium foil at 105°C for 48 h to obtain dry weight. The decomposition over a 2-week period was expressed as % remaining organic material.

### Macrophyte cover, biomass and bioassay

Development of macrophyte species composition and macrophyte species cover was examined three times on days −1, 14, and 44 days. Development of vegetation and the species-composition of macrophytes were investigated by monitoring macrophyte cover and abundance. The monitoring only involved the 75% of the sediment surface that was initially planted. Cover values were estimated using ordinal scales of 1 (<1%), 2 (1–5%), 3 (5–12.5%), 4 (12.5–25%), 5 (25–50%), 6 (50–75%), 7 (75–100%).

At the last sampling date (day 42), aboveground biomass of all macrophyte species were harvested for each microcosm. The plant material harvested was rinsed under tap water to remove sediment particles and macroinvertebrates and then dried in an oven in pre-weighed aluminium foil at 105°C for 48 h to determine the dry weight.

In addition to total macrophyte analysis, a *Myriophyllum spicatum* bioassay was performed. Flower pots (height 9.5 cm: 9 cm diameter) were filled with approximately 8.5 cm depth of sediment consisting of 86% peat, 8% sand, 6% clay and 3.73 kg fertiliser/m^3^ (slow release). Each pot received three apical shoots of *M. spicatum* with a length of 10 cm and with at least one node in the sediment. Only unbranched, non-flowering apical *Myriophyllum* shoots without roots were selected. In the pre-treatment period at day −21, 500 pots were introduced into one of the ditches at the Sinderhoeve Experimental Station. At day −4, 12 pots per microcosm with healthy plants were placed in plastic trays on the macrophyte-free sediment section. On day −3, 16 *M. spicatum* pots (one from each test cosm) were sampled to characterise the plant material (i.e., shoot and root dry weight (105°C for 24 h), shoot length and shoot number) at the time of the first application. On days 14 and 42, 6 pots per microcosm were harvested. The plants were rinsed thoroughly to remove sediment particles. The endpoints (mean per shoot) measured were aboveground dry weight, belowground dry weight (roots), total length of shoots (length of main shoot and length of side shoots), mean length of shoots (total length of shoots/total # of side shoots), and number of side shoots. For each bioassay, belowground material (roots) was separated from the aboveground parts and plant samples were dried in aluminium foil (105°C, 48 h) and weighed.

### Data analysis

#### Univariate analysis

Prior to univariate and multivariate analyses, abundance data of macroinvertebrates, phytoplankton and zooplankton were ln(ax + 1) transformed, where x stands for the abundance value and ax makes 2 by taking the lowest abundance value higher than zero. We deviated from the usual ln(x + 1) transformation because the data set frequently showed low or high abundance values (i.e., 1 individual per substratum for macroinvertebrates, 0.2 individuals per litre for the zooplankton and 2 individuals per litre for the phytoplankton community). We decided that the factor ax in the ln(ax + 1) transformation should make 2 by taking the lowest abundance value higher than zero for x. A factor of two was chosen to avoid false discrepancy between zero abundance values and low abundance values. Since, for instance, the lowest abundance value higher than zero in the zooplankton data sets was 0.2, a factor 10 was used (Van den Brink et al. 2000). All other variables were tested using untransformed values. Statistically significant differences between the treatments as well as against controls were assessed for all parameters or taxon levels at each time point, using analysis of variance (ANOVA) with multiple comparison tests. ANOVA was followed by Duncan’s multiple-range test (*p* < 0.05), testing all treatments against the controls but also against each other. The analyses were carried out with the Genstat computer programme (v11.1, Laws Agricultural Trust, 2009 by VSN International Ltd). If the endpoint was measured more than three times after the initiation of the treatments, effects were only considered when they were consistent, i.e., occurring on least two consecutive sampling dates.

#### Multivariate analysis

**Fig. 2:**
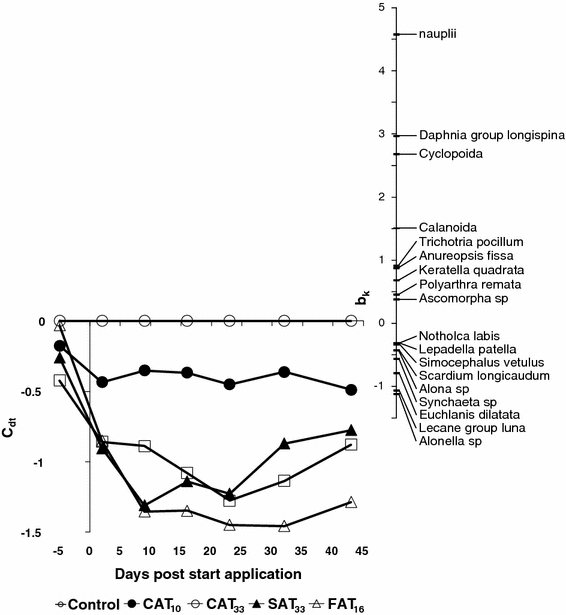
Principal response curves resulting from the analysis of the zooplankton dataset, indicating the effects of different azoxystrobin treatments. Nineteen percent of all variance could be attributed to the sampling date; this is displayed on the horizontal axis. Twenty-six percent of all variance could be attributed to treatment level, 35% of which is displayed on the *vertical axis*. The *lines* represent the development of the treatments in time. The species weight (b_k_) can be interpreted as the affinity of a taxon with the Principal Response Curves (c_dt_). Taxa with a species weight between 0.25 and −0.25 are not shown. A Monte Carlo permutation test indicated that the diagram displays a significant amount of the variance explained by the treatment (*p* = 0.004)

The effects of azoxystrobin treatment at the community level of macroinvertebrates, zooplankton and phytoplankton were analysed by the Principal Response Curves (PRC) method using the CANOCO software package, version 4.5 (Ter Braak and Smilauer 2002; Van den Brink and ter Braak 1999). The analysis results in a diagram showing sampling day on the *x*-axis and the first Principal Component of the treatment effects on the community on the *y*-axis (e.g., Fig. 2). The PRC method yields a diagram showing the most dominant community response to the treatment present in the data set. The species weights are shown in a separate diagram, and indicate the degree of affinity the species have with this dominant response. The results of the PRC analysis can also be evaluated in terms of the fractions of variance explained by the factors time and treatment, and the PRC diagram shows the fraction of the variance that is explained by the treatment.

In the CANOCO computer programme, redundancy analysis is accompanied by Monte Carlo permutation tests to assess the statistical significance of the effects of the treatments on the species composition of the microcosms. The significance of the PRC diagram, in terms of displayed treatment variance, was tested by Monte Carlo permutation of microcosms, using an *F*-type test statistic based on the eigenvalue of the component (van den Brink and ter Braak 1999). For each sampling date, all treatments were also tested against the controls using Monte Carlo permutation tests to assess the significance of treatment effects in time.

## Results

### Exposure to azoxystrobin

Dosing solutions corresponded well (108 ± 8%; mean ± SD) with the nominal concentrations, although measured peak concentrations of azoxystrobin exposure 4 h after the applications were higher than nominal (Table 1). Table 1 shows that the TWA exposure concentrations in CAT_10_ and CAT_33_ were as planned and TWA concentrations in the SAT_33_ and FAT_16_, at 14.9 and 14.7 μg/L, respectively, were almost identical over the period 0–42 days.

**Table 1:** Nominal, peak and time-weighted average (TWA) concentrations (μg/L) of azoxystrobin during the treatment periods (days 0–42)

Treatment exposure regimes	Intended conc. (μg/L)	Nominal initial conc. (μg/L)	% of nominal initial conc. (%)	Measured peak conc. (4-h after application) (μg/L)	% of measured peak conc. (%)	TWA (0–42 days) (μg/L)	% of TWA conc. (%)
CAT_10_	10	11.7	117	12.9	129	9.35	93.5
CAT_33_	33	37.1	112	41.9	127	32.8	99.3
SAT_33_	33	32.8	104	38.1	121	14.9	
FAT_16_	15.8	15.7	99.3	18.4	121	14.7	

Nominal initial concentrations are based on the concentrations measured in treatment solutions

All concentrations are presented as means of three replicates

The dynamics in measured concentrations of azoxystrobin in microcosms treated with different application regimes are illustrated in Fig. 1. In the continuous exposure treatment regimes, the highest measured concentration in CAT_10_ was 14.3 μg/L on day 2.2 and the lowest was 7.0 μg/L on day 16 (Fig. 1a), while the exposure concentration in CAT_33_ was rather higher than target concentration directly after application and fluctuated in the beginning, becoming less variable from day 7 to 42 (Fig. 1b). The highest measured concentrations in CAT_33_ were 41.9, 43.1, and 46.7 μg/L on days 0.17, 1.0 and 2.2, respectively and the lowest was 25.6 μg/L on day 16. At the end of experiment (day 42), the measured azoxystrobin concentration in the SAT_33_ was approximately 20% of the nominal applied (Fig. 1c).The DT_50_ from water-phase as calculated from the concentration dynamics in the SAT_33_ was 18 days. The FAT_16_ shows four pulses followed by slow dissipation between applications (Fig. 1d). After 9 days, exactly one day before the 2nd application in the FAT_16_ treatment, 53% of the initial measured test concentration was still present, while on day 20 and 30, just before the 3rd and 4th application 69 and 71% of subsequent applications were detected, respectively.

### Zooplankton

Over the experimental period, a total of 46 different zooplankton taxa were identified in the microcosms. In terms of total abundance, the zooplankton community was dominated by Rotifera and Copepoda followed by Cladocera and Ostracoda. Rotifera were the most diverse taxonomic group with 33 taxa and 80% of total zooplankton abundance. Four taxa belonged to the *Trichocerca* family making it the most diverse genus, followed by *Lecane* sp. (two species) and *Keratella* sp. (two species). Among the rotifers, *Polyarthra remata* was the dominant species (37% of the total zooplankton abundance), followed by *Synchaeta* sp. (10%), *Keratella quadrata* (9%) and *Hexarthra* sp. (9%). Cladocera were represented by 9 taxa, Copepoda by 3 taxa (copepod nauplii, Cyclopoida, Calanoida) and Ostracoda by 1 taxon (not further identified). Copepod nauplii accounted for 15% of total zooplankton abundance and had a high abundance throughout the experimental period in the controls, with an average of 200 individuals/L.

The Monte Carlo permutation tests indicated that significant treatment-related effects were observed in CAT_33_, SAT_33_ and FAT_16_ (Table 2). The effects of azoxystrobin application on zooplankton community structure are visualised in the PRC diagram presented in Fig. 2. The PRC diagram of the zooplankton data set revealed small non-significant variation in the pre-treatment period but substantial treatment-dependent differences to the controls after the start of the treatments. The zooplankton community response was characterised by pronounced effects in all azoxystrobin treatments except CAT_10_. Up until day 9, the treatment-related responses of the zooplankton community in the SAT_33_ and CAT_33_ treatments, characterised by more or less the same initial peak concentration, were similar, but after that the SAT_33_ treatment showed a trend of recovery in contrast to the CAT_33_ treatment (Fig. 2). Initially (day 9), the treatment-related response of the zooplankton community was more pronounced in the SAT_33_ than in the FAT_16_ treatment, but later on, responses between these treatments (characterised by the same 42-d TWA concentration) were very similar (Fig. 2). The deviation of treatments from the controls was consistent with the results of Monte Carlo tests (Table 2). The high positive species-weight (b_k_ > 1.5) of all Copepoda (copepod nauplii, Cyclopodia, Calanoida) and one Cladocera species (*Daphnia* gr. *longspina*) in the PRC diagram (Fig. 2) indicate that abundances of these taxa correlated best with the community response, herewith showing a treatment-related decline. Several taxa belonging to Rotifera, such as *Lecane* gr. *luna*, *Euchlanis dilatata*, *Synchaeta* sp. and *Scaridium longicaudum*, and the cladocerans *Alonella* sp. and *Alona* sp. had a weak negative species-weight score (b_K_ <−1; Fig. 2), suggesting a small treatment-related increase.

**Table 2:** Results of Monte Carlo permutation tests performed on the zooplankton data set

Day	Control versus CAT_10_	Control versus CAT_33_	Control versus SAT_33_	Control versus FAT_16_	CAT_10_ versus CAT_33_	CAT_10_ versus SAT_33_	CAT_10_ versus FAT_16_	CAT_33_ versus SAT_33_	CAT_33_ versus FAT_16_	SAT_33_ versus FAT_16_
–5										
2		0.03	(0.10)	0.03						
9		0.03	0.03	0.03	(0.09)	(0.09)				
16		0.03		(0.07)						
23		0.03	0.03	0.03	(0.09)					
32		0.03			(0.09)					
43		0.03		0.03						

*p* values between 0.05 and 0.10 are stated between brackets because they are only indicative for significant differences. Empty cells denote *p* values larger than 0.100

**Fig. 3:**
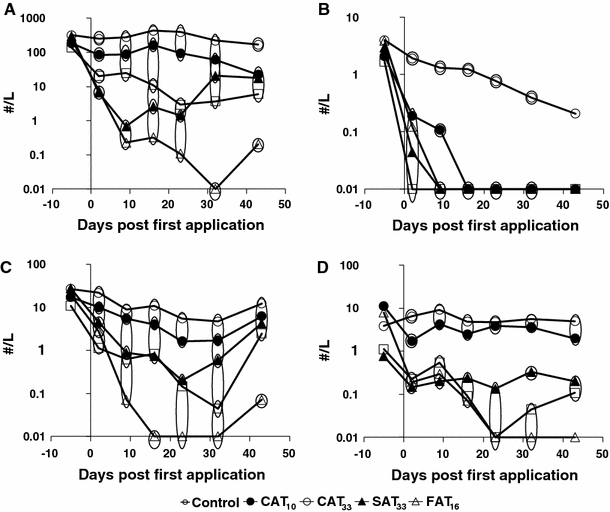
Zooplankton population dynamics, in numbers per litre (geometric mean), of taxa showing consistent responses to azoxystrobin treatments. Nauplii (**a**), Calanoida (**b**), Cyclopoida (**c**) and Daphnia gr. longispina (**d**). Significant differences are indicated by the *circles*. Treatments present in the same *circle* did not differ significantly from each other, while those not sharing the same *circle* did differ significantly (Duncan test, *p* < 0.05). The value 0.01 denotes 0 numbers in the samples

The dynamics of the four taxa that showed consistent statistically significant (Duncan test; *p* < 0.05) treatment related differences in the univariate analyses are shown in Fig. 3. These responses at the taxon level are in accordance with their high species-weight in the PRC diagram. Treatment-related effects on nauplii became apparent soon after application, particularly for the CAT_33_ and SAT_33_ treatments, followed by FAT_16_ and CAT_10_. The most pronounced effects in terms of magnitude and duration were observed for CAT_33_ which was significantly different from other treatments on day 32 and 43 (Fig. 3a). In the course of the experiment, mean densities of nauplii were somewhat lower in the CAT_10_ treatment when compared to controls but for CAT_10_, statistically significant effects were only apparent on two isolated sampling days (day 2 and 43) (Fig. 3a). Again the responses of nauplii in SAT_33_ and CAT_33_ treatment (similar initial peak concentration) were similar up until day 9. At the end of the experiment, densities of nauplii were very similar in the SAT_33_ and FAT_16_ treatments (characterised by similar 42-d TWA concentration). Calanoida disappeared from the FAT_16_ microcosms 2 days after the first application, followed by SAT_33_, CAT_33_ and CAT_10_, respectively (Fig. 3b). Statistically significant differences relative to the controls remained apparent in all treatments throughout the experiment, except for the last sampling date (day 43), which was a result of a decrease of abundance of calanoids in the controls (Fig. 3b). Note that densities of Calanoida already were low in all test systems prior to fungicide application. Cyclopoida showed prominent effects in all treatments except for the treatment CAT_10_ (Fig. 3c). CAT_33_ was significantly different from all other treatments on days 17 and 43 and showed a decline in abundance until the last sampling date. Statistical analysis indicated that partial recovery had occurred in the SAT_33_ and FAT_16_ treatments by the end of the study. No consistent significant effects were detected for CAT_10_ (Fig. 3c). Azoxystrobin had adverse effects on the abundance of the *D.* gr. *longispina* populations in all treatments in the first week after application (Fig. 3d). *D.* gr. *longispina* completely disappeared in CAT_33_ and FAT_16_ after 23 days. Increasing effects were observed after the second application on day 10 of FAT_16_ (Fig. 3d). No recovery was observed in CAT_33_, while partial recovery was observed for SAT_33_ and FAT_16_. At the end of the experiment, densities of *D*. gr. *longispina* were very similar in the SAT_33_ and FAT_16_ treatments (characterised by the same 42-d TWA concentration). For CAT_10_, significant effects could be demonstrated only on day 2 (Fig. 3d).

### Macroinvertebrates

Over the experimental period, a total of 91 different macroinvertebrate taxa were found in the microcosms, which were dominated by Insecta (51 taxa), followed by Mollusca (19), Oligochaeta (7), Hirudinea (5), Turbellaria (5), Crustacea (3) and Hydracarina (1). Among the insects, Diptera and Ephemeroptera accounted for 36 and 14% of total abundance of macroinvertebrates, respectively, of which *Chaoborus obscuripes* and *Cloeon dipterum* were the most abundant taxa. Macrocrustaceans comprised 22% of the total macoinvertebrate abundance and were represented by *Gammarus pulex*, *Asellus aquaticus* and *Proasellus meridianus/coxalis.* Among the non-arthropods, the most abundant taxonomic groups were the Hirudinea and Gastropoda, which accounted for approximately 9 and 11% of total invertebrate abundance, respectively. *Erpobdella* sp. was the most abundant taxon in Hirudinea while *Valvata* sp. was the most abundant taxon in Gastropoda.

**Fig. 4:**
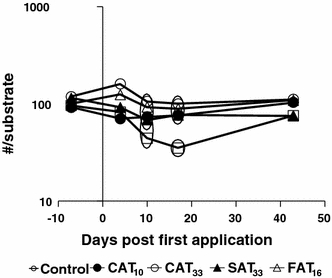
Population dynamics of Chaoborus obscuripes in numbers per substrate (geometric mean). Significant differences are indicated by the *circles*. Treatments present in the same *circle* did not differ significantly from each other, while those not sharing the same *circle* did differ significantly (Duncan test, *p* < 0.05)

At the community level, the PRC analysis of the macroinvertebrate dataset indicated no effects, which was confirmed by the results of the Monte Carlo permutation tests (*p* > 0.05; results not shown). At the species level, statistically significant declines in abundance relative to controls were observed only for *Chaoborus obscuripes* (Fig. 4), in the FAT_16_ treatment in particular. For this treatment, recovery was observed at the end of the experiment (Fig. 4). A similar decline was not observed in the CAT_33_ treatment, characterised by both a higher peak concentration and a higher 42-d TWA concentration than the FAT_16_ treatment.

### Phytoplankton and periphyton

Over the experimental period, a total of 201 different phytoplankton taxa were identified in the microcosms. In terms of numbers of taxa as well as total abundance, the most important taxonomic groups were Chlorophyta (green algae), Charophyta (green algae), Cyanobacteria (blue-green algae) and Bacillariophyta (diatoms). Among the Chlorophyta, the most abundant taxa were *Sphaerocystis* (Tetrasporales; Chlorophyceae) and Chlorophyta 2–5 μm, which accounted for 16% and 3% of the total phytoplankton abundance, respectively. For Cyanobacteria, the most abundant taxa were *Chroococcales* 2–5 μm colony (53%), *Chroococcales* 1–2 μm colony (7%), followed by *Pseudanabaena* (4%) and *Pannus planus* (3%).

The multivariate analysis showed no significant community-level responses to the treatment (Monte Carlo permutation test, *p* > 0.05). In addition, consistent and statistically significant effects at the population level were only detected for *Tetraedron minimum*, which showed a significant reduction in CAT_10_, SAT_33_ and FAT_16_ on the last two sampling dates relative to controls.

Chlorophyll-*a* content of phytoplankton ranged between 0.00 and 36.76 μg/L. For periphyton values ranged between 10.09 and 58.97 μg/cm^2^. No statistical differences between the various treatments and the controls were detected (Duncan test, *p* < 0.05).

### Macrophytes

Over the experimental period, a total of 14 different species of macrophyte were monitored in the microcosms. Rooted submerged macrophyte formed the majority of taxa, comprising of 3 *Potamogeton* species followed by 2 *Elodea* species. The multivariate statistical analysis indicated that the macrophyte community was not significantly affected by azoxystrobin (Monte Carlo permutation test, *p* > 0.05). Univariate analysis of populations indicated statistically significant deviations (Duncan test, *p* < 0.05) for *Spirodela polyrhiza*, by the end of experiment.

**Fig. 5:**
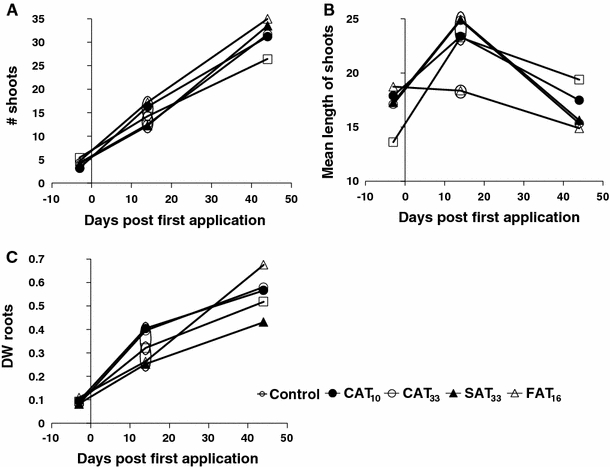
Results of the bioassays performed with Myriophyllum spicatum. Number of shoots (**a**), mean length of shoots (**b**) and weight of roots (**c**). Significant differences are indicated by the *circles* (Duncan test, *p* < 0.05). Treatments present in the same *circle* did not differ significantly from each other, while those not sharing the same *circle* did differ significantly

For the bioassays, 14 days after the start of the azoxystrobin exposures, the number of shoots of *M. spicatum* was significantly higher in CAT_10_ and CAT_33_ compared to the controls (Fig. 5a). The mean length of the shoots was significantly reduced in CAT_33_ at the same sampling time (day 14, Fig. 5b). Significant effects on dry weight of roots were also detected 14 days after the azoxystrobin application in the SAT_33_ and CAT_33_ treatments (Fig. 5c). No consistent significant effects were detected in any treatment on other endpoints (i.e., dry weight of shoots and total length of shoot), nor on the final biomass of macrophyte species (mean dry weight for all cosms = 128 ± 27 g dw/m^2^, mean ± SD).

### Decomposition

No significant effects of the azoxystrobin treatments (Duncan test, *p* < 0.05) were detected in the breakdown of particulate organic matter (POM). The remaining dry weight of leaves in litterbag over the whole experimental period including all microcosms was 80 ± 7% (mean ± SD).

### Water quality analysis

**Fig. 6:**
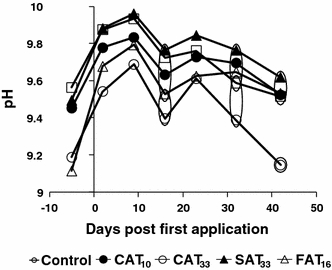
Dynamics of pH in the different treatments. Significant differences are indicated by the *circles*. Treatments present in the same *circle* did not differ significantly from each other, while those not sharing the same *circle* did differ significantly

The water quality variables DO, EC, T and alkalinity did not reveal consistent treatment-related responses and mean values in all microcosms during the entire experimental period were 10.6 ± 1.0 mg/L; 115 ± 14 μS/cm^2^; 19 ± 0.8°C, and 0.84 ± 0.05 meq/L, respectively. An increase in pH was observed for most treatment levels, but kept within one pH unit (Fig. 6). At day 16, pH values were statistically significantly elevated in CAT_10_, SAT_33_ and FAT_16_ while on day 23, in CAT_10_, CAT_33_ and SAT_33_. Notably, deviations of these treatments were statistically significant relative to control rather than from each other (Fig. 6). All treatment regime pH values were significantly different to the control at the end of experiment (Fig. 6).

The concentrations of the ammonia, nitrate and nitrite, and ortho-phosphate were below the LODs of 0.04, 0.03 and 0.02 mg/L, respectively. No significant effects were found on the total phosphate and total soluble nitrogen levels (concentrations between 0.60 and 1.01 mg/L; <LOD and 0.14 mg/L, respectively).

## Discussion

### Comparison across time-variable exposure regimes (similar peak vs. similar 42-d TWA)

To determine which effects can be assessed by the peak and TWA_42d_ exposure concentrations, treatment-related impacts of SAT_33_ and CAT_33_ were compared because they had a similar initial peak concentration (different TWA), and those of SAT_33_ and FAT_16_ are compared because they had similar TWA concentrations (but different peak concentrations). At the community level, the PRC diagram resulting from analysis of zooplankton data-set (Fig. 2) elucidates small and similar sized magnitude of effects among different time-variable exposure profiles (SAT_33_, FAT_16_ and CAT_33_) shortly after the start of the experiment (day 2), while the effects in the SAT_33_ and CAT_33_ increased in magnitude on day 9. The time-variable exposure regimes that have similar initial peak concentrations (SAT_33_ and CAT_33_) resulted in comparable effects on the zooplankton community until day 9, but not afterwards. This indicates that the peak concentration is a good predictor of short-term effects only. The magnitude of effect in SAT_33_ was pronounced relative to FAT_16_ until day 9, after which the magnitude of effects increased in FAT_16_ to become similar to that in SAT_33_ (from day 16 onwards). Since both these pulsed treatment regimes are characterised by the same 42-d TWA concentration, it can be concluded that the similar TWA concentrations cause a comparable effects on the zooplankton community in the long-term. These results support the ELINK recommendation that for long term effects the TWA concentration can be more relevant than the peak concentration (Brock et al. 2010). During the last few years, several workshops and projects have proposed using the TWA concentration approach, instead of the nominal peak concentration for assessing effects of repeated exposures (ECOFRAM 1999; Boxall et al. 2001; Reinert et al. 2002; Boesten et al. 2007; Brock et al. 2010).

The finding that TWA is a better predictor of effects also holds true at the taxon level. Several taxa such as nauplii, adult Cyclopoida and *Daphnia* gr. *longispina* clearly show similar survival responses to the different time-variable exposure regimes (Fig. 3a, c and d), i.e., similar effect magnitude for the SAT_33_ and CAT_33_ treatments (similar peak concentration) during the first 1.5 week after the first application and similar effect magnitudes for the FAT_16_ and CAT_33_ treatments (similar TWA concentrations) at the end of the experiment.

For the risk assessment of azoxystrobin, it is not so surprising that the short-term effects observed due to different peak concentrations in the microcosm experiment can be related to measured or predicted peak concentrations. The results suggest that long-term effects can be assessed by comparing TWA rather than peak concentrations, even if the dynamics of the pulses are different. Zafar et al. (2011) also found similar relationships between long-term effects and TWA concentrations for most invertebrate species exposed to chlorpyrifos in microcosm under different exposure profiles with the same TWA concentration. They also concluded that for the applied combination of concentration dynamics, the TWA concentration was a more adequate predictor for long-term risks of chlorpyrifos for most species than the peak concentration. After performing long-term toxicity tests with *Gammarus. pulex*, Ashauer et al. (2007) also concluded that the TWA concentration approach can be used to predict effects of repeated pulses of chlorpyrifos and pentachlorophenol.

### Fate of azoxystrobin in the water column

The dissipation rate of azoxystrobin in the SAT_33_ treatment of the present study (18 days) is consistent with those of previous studies, which reported half-life values in the range of 15–25 days (Gustafsson et al. 2010) and 13 days (Jones and Lake 2000). The dissipation is probably a result of photolysis, since the US-EPA (1997) reported a half-life of 11–17 days in aquatic environments for photolysis only. Also, the potential for accumulation in sediments is low (log K_ow_ = 2.50) and azoxystrobin is a non-volatile compound (Henry’s Law constant = 7.3 × 10^−9^ Pa × m^3^/mol (Tomlin 2011). The higher peak concentration in all treatments measured 4 h after application compared to the nominal concentrations may be attributed to non-homogeneity of azoxystrobin in the water layer as a result of dominant aquatic vegetation and therefore incomplete mixing of dosed solutions within the microcosm.

### Biological effects of azoxystrobin

The PRC diagram of the zooplankton community indicated pronounced treatment dependent negative impacts of azoxystrobin (Fig. 3). The largest adverse effects were reported for nauplii, adult Calanoida and Cladocera, followed by adult Cyclopodia. For most taxa these changes persisted until the end of study (Figs. 2, 3). These results are consistent with the other model ecosystem studies available and suggest that some copepods are sensitive to azoxystrobin (Cole et al. 2000; Gustafsson et al. 2010). Furthermore, in the present study, naupliar stages of copepods were found to be more sensitive to azoxystrobin than adult cyclopoids, which is in agreement with observations reported by Gustafsson et al. (2010).

The decrease in numbers of calanoid copepods in all treatments just after the start of the treatment (Fig. 3b) is similar to other observations by Lauridsen et al. (2003). They performed a series of acute and sub-chronic toxicity tests with azoxystrobin on several different freshwater zooplankton and macroinvertebrate species and found that the calanoid copepod *Eudiaptomus graciloides* was the most sensitive among the tested taxa. In the present study, cyclopoid copepods were vulnerable to all treatments except for CAT_10_ (Fig. 3c). This is not in line with the observations by Cole et al. (2000) who reported effects directly after a single application of 10 μg/L. This may be a result of differences between the species composition of the Copepoda community. The effects in this study agree with the laboratory tests performed by Lauridsen et al. (2003) with the cyclopoid copepod *Cyclops vicinus*, in which all individuals died within 48 h when exposed to 20 μg/L or higher, and a NOEC for reproduction of 10 μg/L was reported. Effects on Copepoda populations have also been reported longer than 3 weeks period on Copepoda populations after a single application of 3, 7.5, 15, and 60 μg/L (Gustafsson et al. 2010).

The PRC diagram and univariate analysis showed a negative impact of azoxystrobin on one taxon of Cladocera, i.e., *D.* gr. *longispina*, while other taxa like *Simocephalus vetulus*, *Alona* sp. and *Alonella* sp. experienced no effects (Fig. 2). This is in accordance with the study of Cole et al. (2000), who reported significant reductions of *Daphnia* spp. after single applications of 10 and 30 μg/L and also reported significant increase in numbers for *Chydorus* sp. It was shown by Lauridsen et al. (2003) that some cladocerans (*D. magna*, *Chydorus sphaericus* and *Ceriodaphnia* sp.) were relatively tolerant to azoxystrobin while others (*D. galeata*) were much more sensitive. Their physiological experiments (e.g., pectoral limb, hind claw, mandible and heart activity) clearly demonstrated that azoxystrobin may affect zooplankton in different ways.

Treatment-related impacts on the macroinvertebrate community were not found. On the basis of information already known for azoxystrobin, it is reasonable to assume that invertebrates, in particular macroinvertebrate crustaceans and insects, are not highly sensitive to azoxystrobin, which is supported by the results of Cole et al. (2000). *C. obscuripes* was the only macroinvertebrate species which responded significantly to the FAT_16_ treatment (Fig. 4). The observed effect in the FAT_16_ treatment is probably not a direct effect since it did not show a clear treatment-response pattern. The effects observed are consistent with the microcosm study conducted by Cole et al. (2000) who reported no significant effects on any macroinvertebrates after single applications of 10 and 30 μg/L and observed some effects on Gammaridae and Mollusca at 100 μg/L. Lauridsen et al. (2003) reported no effects on *Chaoborus flavicans* up to azoxystrobin concentrations of 6,000 μg/L and no significant effects were detected on Chaoboridae after a single application of 1,000 μg azoxystrobin/L in the microcosm study performed by Cole et al. (2000). The observed effects might be a result of temporally decreased food availability in the form of *D.* gr. *longispina* (Fig. 3), while its fast recovery can be explained by the multivoltine life cycle of *C. obscuripes* in The Netherlands (Van Wijngaarden et al. 2006). Further studies performed by Lauridsen et al. (2003) on three other macroinvertebrate species, i.e., *Chironomus plumosus*, *Cloeon dipterum* and *Hydropsyche angustipennis,* also revealed no effects at treatment levels of 525, 1,500 and 3,000 μg/L, respectively. Apparently, the sensitivity-tolerance spectrum for this chemical is very wide for different arthropod species.

Compared to the controls, the composition of the phytoplankton community was not altered by the azoxystrobin treatments, which is in agreement with Cole et al. (2000). On day 22, the abundance of *Tetraedron minimum* had increased in the control and the CAT_33_ treatments, and abundance was still elevated at day 42 in these treatments. It is difficult to explain the significantly lower numbers in the other treatments relative to the control, as a result of direct or indirect effects of azoxystrobin as effects were not observed in the treatment with the highest peak and TWA concentration (i.e., CAT_33_). A significant increase of the macrophyte *Spirodela polyrhiza* coverage occurred in the CAT_33_, SAT_33_ and FAT_16_ treatments in the range of % cover of <1% and is, therefore, not considered important from ecological point of view.The absence of systematic effects on macrophytes in this study (Fig. 5) is in agreement with the high growth EC_50_ (3,200 μg/L) determined for the macrophyte *Lemna gibba* in a laboratory toxicity study (Tomlin 2011). As a result of this lack of effects on macrophytes, the water physico-chemical parameters were not greatly influenced by azoxystrobin treatments. Of all the physico-chemical variables, pH values in treated systems were slightly (and sometimes statistically significantly) higher than in the controls (Fig. 6). This could be a result of increased algal biomass due to a reduced grazing pressure by *D.* gr. *longispina*, but this was, however, not reflected in significant increases in phytoplankton and periphytonic chlorophyll-*a*. The observations in the present study are in accordance with Gustafsson et al. (2010) who found marginally significant differences on one sampling day for chlorophyll-*a* concentration in water at azoxystrobin concentrations of 5 and 20 μg/L, although it should be noted that this experiment was performed in brackish water. The increase in pH values remained within one pH unit and are, therefore, considered to be of low ecological relevance. In line with this, Cole et al. (2000) also found significant differences in pH relative to the control, following a single application of 10 μg/L, but on individual sampling dates only. No effects were observed on decomposition in all treatment regimes which is consistent with Gustafsson et al. (2010) who reported that degradation of organic material in their microcosms was not affected by azoxystrobin treatment during the course of experiment.

According to the EU Guidance Document on Aquatic Ecotoxicology, the no observed ecologically adverse effect concentrations (NOEAEC) is the concentration at or below which no long-lasting adverse effects were observed in the microcosm study (European Commission 2002). If we translate short-lasting effects to effect class 1 or 2 (based on effect classes; Brock et al. 2006), the NOEAEC of this study can be set at the CAT_10_ treatment, which is in accordance with the NOEAEC of a previous microcosm study (Cole et al. 2000) which was set at a single application of 10 μg/L.

## Conclusions

The present study shows that under the tested exposure regimes and for the endpoints studied, the TWA is a more adequate predictor for long-term effects of azoxystrobin on the zooplankton community and species than the peak concentration. These results support the recommendation of the ELINK workshop that for long-term effects of pesticides, a risk assessment based on TWA concentrations may be more relevant than one based on peak concentrations. It should be noted however that this conclusion only applies to the zooplankton community and species, since effects on other endpoints were limited.

## References

[CR1] Ashauer R, Boxall ABA, Brown CD (2007). Modeling combined effects of pulsed exposure to carbaryl and chlorpyrifos on Gammarus Pulex. Environ Sci Technol.

[CR2] Bartlett DW, Clough JM, Goodwin JR, Hall AA, Hamer M, Parr-Dobrzanski B (2002). The strobilurin fungicides. Pest Manag Sci.

[CR3] Boesten JJTI, Köpp H, Adriaanse PI, Brock TCM, Forbes VE (2007). Conceptual model for improving the link between exposure and effects in the aquatic risk assessment of pesticides. Ecotoxicol Environ Saf.

[CR4] Boxall A, Brown C, Barrett K (2001) Higher tier laboratory aquatic toxicity testing. Cranfield centre for EcoChemistry research report No. JF 4317E for DETR, Canfield

[CR5] Brock TCM, Huijbregts CAM, Van de Steeg-Huberts MJ, Vlassak MA (1982). In situ studies on the breakdown of Nymphoides peltata (Gmel.) O. Kuntze (Menyanthaceae); Some methodological aspects of the litter bag technique. Hydrobiol Bull.

[CR6] Brock TCM, Crum SJH, Wijngaarden R, Budde BJ, Tijink J, Zuppelli A, Leeuwangh P (1992). Fate and effects of the insecticide Dursban^®^ 4E in indoor Elodea-dominated and macrophyte-free freshwater model ecosystems: I. Fate and primary effects of the active ingredient chlorpyrifos. Arch Environ Contam Toxicol.

[CR7] Brock TCM, Arts GHP, Maltby L, Van den Brink PJ (2006). Aquatic risks of pesticides, ecological protection goals and common aims in EU Legislation. Integr Environ Assess Manag.

[CR8] Brock TCM, Alix A, Brown CD, Capri E, Gottesbüren BFF, Heimbach F, Lythgo CM, Schulz R, Streloke M (2010). Linking aquatic exposure to effects: risk assessment of pesticides.

[CR9] Cole JFH, Everett CJ, Gentle W, Ashwell JA, Goggin U (2000) Azoxystrobin: an outdoor pond microcosm study. ZENECA Agrochemicals. Jealott’s Hill International Research Centre. Report number RJ2857B, Bracknell (summarised and cited in EFSA 2009)

[CR10] ECOFRAM (1999). Ecological committee on fifra risk assessment methods (ECOFRAM) ECOFRAM draft aquatic report.

[CR11] EFSA (2009) Council directive 91/414/EEC. Azoxystrobin. Report and proposed decision of the United Kingdom made to the European Commission under commission regulation 737/2007. Accessed via http://dar.efsa.europa.eu/dar-web/provision

[CR12] European Commission (2002) Guidance document on aquatic toxicology in the context of the Directive 91/414/EEC. Working Document of the European Commission Health & Consumer Protection Directorate-General, Brussels

[CR13] European Commission (2009). Regulation (EC) No 1107/2009 of the European Parliament and of the Council of 21 October 2009 concerning the placing of plant protection products on the market and repealing Council Directives 79/117/EEC and 91/414/EEC. Off J Eur Comm.

[CR14] Gustafsson K, Blidberg E, Elfgren I, Hellström A, Kylin H, Gorokhova E (2010). Direct and indirect effects of the fungicide azoxystrobin in outdoor brackish water microcosms. Ecotoxicology.

[CR15] Jones RN, Lake A (2000) Azoxystrobin: distribution in an outdoor pond. ZENECA Agrochemicals Report no. RJ 3062B, Bracknell (summarised and cited in EFSA 2009)

[CR16] Lauridsen TL, Friberg-Jensen U, Cristoffersen K (2003) Effekter af cypermethrin, azoxystrobin og bentazon på limniske invertebrater. Rep. 76, Miljøstyrelsen, Miljøministeriet (in Danish), Copenhagen

[CR17] Maltby L, Brock TCM, Van den Brink PJ (2009). Fungicide risk assessment for aquatic ecosystems: importance of interspecific variation, toxic mode of action, and exposure regime. Environ Sci Technol.

[CR18] Reinert KH, Giddings JM, Judd L (2002). Effects analysis of time-varying or repeated exposures in aquatic ecological risk assessment of agrochemicals. Environ Toxicol Chem.

[CR20] Slijkerman DME, Baird DJ, Conrad A, Jak RG, van Straalen NM (2004). Assessing structural and functional plankton responses to carbendazim toxicity. Environ Toxicol Chem.

[CR21] Ter Braak CJF, Smilauer P (2002) CANOCO reference manual and CanoDraw for windows user’s guide: software for canonical community ordination (version 4.5). Microcomputer Power, Ithaca (www.canoco.com)

[CR22] Tomlin CDS (2011). The pesticide manual world compendium.

[CR23] US-EPA (1997) Pesticide tolerance petition filing for azoxystrobin. Federal Register Document 97-5683. Mar 11, 1997

[CR24] Van den Brink PJ (2008). Ecological risk assessment: from book-keeping to chemical stress ecology. Environ Sci Technol.

[CR25] Van den Brink PJ, Ter Braak CJF (1999). Principal response curves: analysis of time-dependent multivariate responses of biological community to stress. Environ Toxicol Chem.

[CR26] Van den Brink PJ, Hattink J, Bransen F, Van Donk E, Brock TCM (2000). Impact of the fungicide carbendazim in freshwater microcosms. II. Zooplankton, primary producers and final conclusions. Aquat Toxicol.

[CR27] Van Wijngaarden RPA, Brock TCM, Van den Brink PJ, Gylstra R, Maund S (2006). Ecological effects of spring and late summer applications of Lambda-Cyhalothrin on freshwater microcosms. Arch Environ Contam Toxicol.

[CR28] Warming T, Mulderij G, Christoffersen K (2009). Clonal variation in physiological responses of Daphnia magna to the strobilurin fungicide azoxystrobin. Environ Toxicol Chem.

[CR29] Webb DJ, Burnison BK, Trimbee AM, Prepas EE (1992). Comparison of chlorophyll a extractions with ethanol and dimethyl sulfoxide/acetone, and a concern about spectrophotometric phaeopigment correction. Can J Fish Aquat Sci.

[CR30] Zafar MI, Van Wijngaarden RPA, Roessink I, Van den Brink PJ (2011). Effects of time-variable exposure regimes of the insecticide chlorpyrifos on freshwater invertebrate communities in microcosms. Environ Toxicol Chem.

